# Pathway-Driven Approaches of Interaction between Oxidative Balance and Genetic Polymorphism on Metabolic Syndrome

**DOI:** 10.1155/2017/6873197

**Published:** 2017-01-16

**Authors:** Ho-Sun Lee, Taesung Park

**Affiliations:** Interdisciplinary Program in Bioinformatics and Department of Statistics, Seoul National University, Seoul 152-742, Republic of Korea

## Abstract

Despite evidences of association between basic redox biology and metabolic syndrome (MetS), few studies have evaluated indices that account for multiple oxidative effectors for MetS. Oxidative balance score (OBS) has indicated the role of oxidative stress in chronic disease pathophysiology. In this study, we evaluated OBS as an oxidative balance indicator for estimating risk of MetS with 6414 study participants. OBS is a multiple exogenous factor score for development of disease; therefore, we investigated interplay between oxidative balance and genetic variation for development of MetS focusing on biological pathways by using gene-set-enrichment analysis. As a result, participants in the highest quartile of OBS were less likely to be at risk for MetS than those in the lowest quartile. In addition, persons in the highest quartile of OBS had the lowest level of inflammatory markers including C-reactive protein and WBC. With GWAS-based pathway analysis, we found that VEGF signaling pathway, glutathione metabolism, and Rac-1 pathway were significantly enriched biological pathways involved with OBS on MetS. These findings suggested that mechanism of angiogenesis, oxidative stress, and inflammation can be involved in interaction between OBS and genetic variation on risk of MetS.

## 1. Introduction

Oxidative stress is a complex and multifactorial process that results from an imbalance between antioxidant protection and reactive oxygen species produced by prooxidants [1]. Basic research has established a link between oxidative stress and pathogenesis of human illness, including cardiovascular disease and cancer with the process of aging [2]. Despite the solid molecular and mechanistic theory of oxidative stress and its role in chronic disease, most clinical trials and observational studies failed to provide a clear answer to the effects on chronic disease by individual prooxidant or antioxidant.

Studies of diet and health have demonstrated that combination of antioxidant factors can be more strongly associated with disease risk than any single nutrient [3]. In this way, it appears possible that a combination of oxidative stress-related factors may be more strongly associated with health outcomes than any individual exposure [4]. Therefore, several groups previously proposed an oxidative balance score (OBS) as a measure of combined pro- and antioxidant exposure status, indicating the role of oxidative stress in human chronic disease pathophysiology and found that OBS can be a comprehensive oxidative balance marker [5–7]. OBS has been shown to correlate inversely with several cancers including colorectal adenoma and prostate cancer and chronic kidney diseases [5, 8, 9]. Nevertheless, the exact mechanism of how the combination of pro- and antioxidants influences diseases is still not fully understood.

The number of individuals with metabolic syndrome (MetS) is increasing worldwide. It was reported that MetS affects 20% to 30% of the population in developed countries [10]. MetS is of substantial clinical relevance and concern because of its high prevalence and association with the development of more serious pathologies. Additional to the health issues directly associated with MetS, this condition contributes to a 5-fold increased risk of type 2 diabetes (T2D), 3-fold increased risk of cardiovascular disease, and also an increased risk of developing common cancers of liver, colorectal, bladder, and so on [10, 11]. Growing evidences suggest that the pathogenic role of oxidative stress causes cellular damage and dysfunction which are associated with MetS and clinical complications [12]. The link between oxidative stress and MetS can be accepted because there is the association of the end-products of free radical-mediated oxidative stress with body mass index (BMI), insulin resistance, hyperlipidemia, and hypertension. In addition, a number of studies indicate that oxidative stress along with chronic inflammatory condition paves the way for the development of MetS-related manifestations, including atherosclerosis, hypertension, and T2D [13, 14]. Despite evidences of association between basic redox biology and MetS, few studies have evaluated combination of oxidative score or indices that account for multiple oxidative effectors with MetS.

It is well accepted that not only exogenous factors including diet and smoking but also endogenous factors like genetic variation contribute to the development of MetS. Genome-wide association studies (GWAS) have identified important susceptible loci [15] that are significantly associated with the risk of MetS. Nevertheless, these findings explain only a small portion of the heritability and clear association of genetic factors with causative effects on MetS requires further elucidation.

Genetic variation also modulates reactive oxidative stress, which may influence several diseases through their interaction with OBS components [16]. Slattery et al. pointed the importance of interaction between OBS and endogenous factors for risk of diseases [16, 17]. For better understanding of the comprehensive mechanism of oxidative balance for the risk of MetS, it has to consider interaction effect between OBS and genetic variation for development of diseases. The identification of genes-environment interaction related to oxidative stress has been studied for cardiovascular disease, atherosclerosis, diabetes, and so on [18, 19]. However, these studies focused on only a few SNPs or selective SNPs with environment factors and considered each of the identified SNPs independently; therefore, it was hard to explain comprehensive action of multiple genes or biological processes in complex diseases. As an alternative to GWAS-driven approaches to detect interaction effect, pathway approach may help identify potential biological pathways that might be missed by the standard GWAS approach [20, 21].

The purpose of this study is to investigate the relationship between OBS and risk of MetS. First, we used OBS as an oxidative balance indicator for estimating risk of MetS. OBS is multiple exogenous factor score for development of disease; therefore, we investigated interplay between oxidative balance and genetic variation for development of MetS. We focused on biological pathways by using gene-set-enrichment analysis (GSEA) for investigating how OBS affects development of MetS with genetic factors.

## 2. Material and Methods

### 2.1. Study Participants

This study was based on the Korea Association Resource (KARE) data included in the Korean Genome Epidemiology Study (KoGES). The details of the study design and procedures used in the KoGES have been described previously [22]. The population based cohort study included 10,030, aged ≥40 years, at baseline from 2001 to 2002. This present analysis used cross-sectional data from 2007 to 2008, the samples were scrutinized for quality control purposes, and 6,688 participants were remained. Study participants completed a questionnaire on sociodemographic status, lifestyle, and disease history. Participants completed self-administered lifestyle and food frequency questionnaires (FFQ), which assessed nutrient intake over the past year of 166 food items. Anthropometrics and biochemical measurements were also described by the previous study [22]. We excluded 261 participants with missing data on at least one MetS component and 115 participants with missing data on at least one OBS component. We also excluded 79 participants with missing data for key covariates such as geographic area, age, and sex. Therefore, we used data for 6414 study participants for final analyses. Written informed consent was obtained from all participants at the KoGES and this research project was approved by the Institutional Review Board of Korea National Institute of Health.

### 2.2. Definition of Metabolic Syndrome and Related Disorders

MetS is characterized by the clustering of several components including abdominal obesity, hypertension, dyslipidemia, insulin resistance, and glucose intolerance that are important precursor of cardiovascular disease and type 2 diabetes. MetS was defined by the presence of three or more of the following five components according to the NCEP-ATP III criteria [23], except for the determination of central obesity. Waist circumference cut-off value was used based on the report by the Korean Society for the Study of Obesity [24]: (1) central obesity given as waist high circumference (≥90 cm for men and ≥85 cm for women), (2) high concentration for serum triglycerols (≥150 mg/dL), (3) low concentrations of serum HDL cholesterol (<40 mg/dL for men and <50 mg/dL for women), (4) hypertension (systolic/diastolic pressure ≥ 130/85 mmHg) or antihypertensive medication, and (5) high concentrations of fasting glucose (≥100 mg/dL) or antidiabetic medication.

Diabetes was diagnosed in subjects who had any one of the following: (1) fasting plasma glucose ≥ 126 mg/dL; (2) postprandial 2 h plasma glucose ≥ 200 mg/dL after a 75 g OGTT; and (3) HbA1c ≥ 6.5% [25]. Subjects who were using insulin or oral antidiabetic drugs at baseline were also defined as having diabetes.

### 2.3. OBS and OBS Weighting

The OBS was calculated by combining information from a total of 7 a priori selected pro- and antioxidant factors, including three prooxidant (smoking, alcohol consumption, and total iron intake) and four antioxidant exposures (*β*-carotene, vitamins C, retinol, and physical activity). The continuous variables reflecting prooxidant and antioxidant exposures divided into low, medium, and high categories based on each exposure's tertile values and points (0 to 2) were given according to tertile categorization. Prooxidants were scored in the opposed way to the antioxidants. A higher score represents a predominance of antioxidant exposures over prooxidant exposures. In our study, OBS is the sum of tertile (Q1 = 0; Q2 = 1; Q3 = 2) of intake of vitamin C, retinol and carotene, and physical activity (0–72.7 MET/day = 0; 72.8–92.4 MET/day = 1; 92.5 ≥ MET/day = 2; MET, metabolic equivalent) with antioxidant components. OBS included smoking status (never = 2, former = 1, and current = 0) and alcohol consumption (nonregular drinker, 0–5 g/day = 0; regular drinker, 5 ≥ g/day = 2). Evaluations of individual OBS component according to quartile of OBS-equal weight (equal weighting of the selected components and a simple summation) were presented in Table 1.

Depending on contribution of OBS components on oxidative balance, we scored OBS with 2 additional weighting methods besides equal weighting method. For beta coefficient weighting, the coefficient estimates for each of the components obtained from the regression model were used to calculate weights for OBS-beta coefficient. Coefficients were multiplied by −1 so that components inversely associated with metabolic disease risk had positive weight and vice versa. Principal component analysis (PCA) was a weight method using variation reduction [26]. The number of PCs to retain was determined by using the diagram of eigenvalues and the interpretability of the PCs. The PC score for each pattern was constructed by summing up and observed OBS components weighted by the PC loading. For weighting of OBS, each model was adjusted for age, sex, geographic area, and BMI.

### 2.4. Genotyping

The KARE dataset consists of the individual SNP chip genotypes and the epidemiological/clinical phenotypes for studying the genetic components of Korean public health. DNA samples were isolated from the peripheral blood of all participants and were genotyped with Affymetrix Genome Wide Human SNP array 5.0 (Affymetrix Inc, Rockville, MD, USA). The obtained KARE dataset passed the quality control criteria and was reported in previous GWAS reports [27]. After sample and genotype quality controls, 344,396 SNPs for 6,414 individuals were available in the KARE data.

### 2.5. Pathway-Based Analysis

It has now been recognized that a majority of biological behaviors are manifested from a complex interaction of biological pathway. Pathway-based approaches using GWAS data are now used routinely to study complex disease like MetS [28, 29]. To analyse pathway involved in OBS and genome-wide interaction, we used the improved i-GSEA that estimates the pathway-level interactions between genetic variants and OBS.

In our study, 344,396 SNPs were mapped to genes with 100 kb boundaries and pathways with <20 genes or >200 genes were excluded from further analysis to reduce the multiple-testing issue and to avoid testing overly narrow or broad functional categories [30]. A false-discovery rate (FDR) was used for multiple-testing correction with <0.05 considered to be significant. Improved GSEA uses a comprehensive pathway/gene set database from SNP data with pathways integrated and curated from a variety of resources, including KEGG (Kyoto Encyclopedia of Genes and Genomes pathway database), Biocarta, and GO (gene ontology) database [31].

### 2.6. Statistical Analysis

OBS components except for physical activity were not normally distributed, so they were log-transformed when assigning OBS. Tests for linear trend were performed using a sum of scores with values from Q1 to Q4, consistent with the quartile grouping. Each OBS weighting method was divided into quartiles, with the lowest quartile (predominance of prooxidants) used as reference. Even though obesity is a main factor of oxidative stress, BMI is a strong risk factor for MetS; therefore, we remove it from OBS components but controlled for it in the statistical models.

Logistic regression analyses were used to examine the relationship between OBS and incidence of MetS adjusting for age, geographic area, sex, and BMI. For comparing the effect of different weighting methods, receiver operating characteristics (ROC) curves and the respective areas under the curves (AUCs) were calculated.

For investigating relationship between OBS and inflammation, we examined the association of OBS with CRP (C-reactive protein) and WBC (white blood cell count). CRP was not normally distributed, and so it was log transformed. The results of the linear regression models were expressed as regression coefficients and their corresponding 95% confidence intervals adjusted for same potential confounding factors as the previous ones.

For elucidating biological process through interaction of gene and oxidative stress by pathway analysis, we used to test gene-environment interaction by performing a 1df test of *H*_0_: ß_gobs_ = 0 (The context of the linear model: log⁡[*p*/(1 − *p*)] = ß_0_ + ß_age_Age + ß_sex_Sex + ß_area_Area + ß_obs_OBS + ß_bmi_BMI + ß_g_G + ß_gobs_G*∗*OBS). Due to the biological contributions to oxidative balance, we used OBS by beta coefficient weight. The interaction term was used to assess the significance of the interaction between genetic variants and OBS. The critical *p* values for accessing the significance of interaction were calculated by FDR *q*-value, at the 5% level. All statistical analyses were performed using R Statistical Software (version 2.14.0; R Foundation for Statistical Computing, Vienna, Austria).

## 3. Results

### 3.1. Baseline Characteristics

A total of 6414 participants (mean age, 56.07 years, SD, 8.29 years) were included in this study. The distribution of characteristics according to quartile of OBS-equal weight is shown in Table 2. Compared to those in the lowest OBS quartile, participants in the highest quartile were likely to be younger and more educated and had more female. Participants in the highest OBS quartile were also tended to have a higher income and reside in rural area and higher total energy intakes than those in the lowest quartile. There was no significant difference in BMI across OBS intervals.

### 3.2. OBS Components and Their Assessment with Metabolic Syndrome

As expected, intake of antioxidants including vitamin C, carotene, and retinol was higher among participants with higher OBS values (data not shown). Contrary to expectation, intakes of iron were higher in the upper OBS quartile group. Participants in the higher OBS quartiles were also more likely to never be smokers, nonregular drinkers, and higher physical activity (data not shown). Individual component level of OBS between non-MetS and MetS was provided in Table 3. In non-MetS patients, levels of dietary components were higher in the highest quartile of OBS. We found that higher consumption of alcohol in a day was observed in non-MetS patients although alcohol is a prooxidant component.

We firstly confirmed that there is no association between each OBS component and MetS (data not shown). For categorical analyses, participants in the highest quartile of all three OBS by weighting methods were less likely to be at risk for MetS than those in the lowest quartile, with statistical significance (Table 4). We observed approximately 40% lower risk of MetS at the highest quantile of OBS compared to reference quantile (lowest quantile) among study participants by beta coefficient adjusted by area, sex, age, and BMI. For OBS-equal weight and OBS-PCA weight, there was around 28–32% reduction in risk of MetS. We used ROC curve for comparing each weighting method for MetS and found there was no difference of AUCs by different weighting methods: OBS-equal weight, beta coefficient weight, and PCA weight (AUC 0.823, 0.824, and 0.824, respectively). We also investigated association between OBS and the odds ratio of MetS without T2D patients. As a result, there is positive association between OBS and MetS regardless of T2D statues.

### 3.3. Association of the OBS and Metabolic Related Factors

Table 5 showed the association between OBS and metabolic related factors. Among components of MetS, ratio of hypertriglyceridemia and low HDL cholesterol had inverse correlation with OBS (*p* trend < 0.01). Persons in the highest quantile of OBS had the lowest level of MetS score, CRP, and WBC.

Oxidative stress and inflammation have been associated with MetS and oxidative stress can increase inflammation and vice versa. Confirming the role of OBS as oxidative stress indicator, we tested association of OBS with inflammatory markers. The CRP and WBC in blood serve as inflammatory markers although these markers have nonspecific features [32]. The regression beta coefficients indicate that OBS was negatively associated with CRP and WBC (Table 6). Compared to the reference group (using the lowest interval as reference), the highest group was negatively correlated with CRP, and all three groups were negatively correlated with WBC.

### 3.4. Pathway Interacted with OBS and Genotype for Metabolic Syndrome

With 352,228 SNPs having *p* values for interaction term in the logistic regression model, we first obtained the significance of the interaction between genetic variants and OBS at SNP level. Then, we tested pathway-based interaction between OBS and genetic variation with *p* values of SNPs for enriched biological processes in MetS.

To conduct the biological pathway analysis, we used all SNPs that were used in GWAS analysis. As shown in Table 7, we found that three canonical pathways (2 were from 190 KEGG pathways and 1 was from 260 Biocarta pathways) were significantly enriched with FDR < 0.05 for MetS, and other 2 pathways were possibly associated with MetS, with FDR < 0.10. The most significant pathway was VEGF signaling pathway (nominal *p* < 0.001, FDR = 0.020). Other significant pathways were glutathione metabolism (nominal *p* < 0.001, FDR = 0.022) and Rac-1 pathway (nominal *p* < 0.001, FDR = 0.045). These findings suggested that pathways of angiogenesis, oxidative stress, and inflammation can be involved in interaction with OBS on MetS risk.

## 4. Discussion

In this study, we examined the possibility of OBS as a predictor of MetS risk and found that higher OBS, which indicates predominance of antioxidant exposures, was associated with significant reduction of the risk of MetS. Considering different contribution of each component on risk of MetS, we used different weighting scheme for combination of pro- and antioxidant exposure into a single score. Dash et al. mentioned that combination of antioxidants and prooxidants into a single score may be more powerful measurement of oxidative stress than approaches that use a single antioxidant or prooxidant [33]. Several studies using OBS also concerned about using an equal weighting method for scoring of oxidative stress. We found that associations between OBS and the risk of MetS were not different depending on OBS weight methods.

CRP and WBC are inflammation markers which have been shown in multiple prospective epidemiological studies to predict the risk of cardiovascular disease and MetS [34]. We found that OBS was inversely associated with inflammatory markers of CRP and WBC, suggesting a role of OBS in the pathogenesis of MetS. High-sensitivity CRP, a marker of low-grade systemic inflammation, is reported as an independent risk factor of diabetes and cardiovascular disease [35]. WBC is also a routinely measured marker of systemic inflammation and is reported to be a risk factor of cardiovascular disease and MetS [36, 37]. Oxidative stress and inflammation are closely related pathophysiological processes, one of which can be easily induced by another. Kong et al. recently reported that OBS was inversely correlated with plasma concentrations of F2-IsoPs, an oxidative stress marker, and CRP levels in persons with colorectal adenoma [9]. We did not compare OBS with any oxidative stress markers that influenced directly oxidative cellular damages, but, we confirmed that OBS is closely associated with inflammation.

Pathway analysis on MetS focuses on the combined effects of multiple SNPs within a gene and multiple genes within a pathway that are grouped according to their shared biological function. Current GWAS-derived pathway analysis can provide insights into mechanism of disease and biological pathways considering interaction between genes and environment factors [38, 39]. We identified that oxidative balance was significantly associated with the risk of MetS in this study. Oxidative balance may interact with polygenic factors in complicated ways to influence MetS susceptibility. For precise biological mechanism of oxidative balance for the risk of MetS, we considered the interaction between endogenous factor and OBS which suggested the comprehensive biological pathways which might modify the risk of MetS. Of the 15,576 genes mapped, 3 pathways had enrichment scores better than FDR < 0.05 (Table 7): VEGF pathway, glutathione metabolism, and Rac-1 pathway which are involved in oxidative stress, angiogenesis, or inflammation [2, 40, 41].

VEGF (vascular endothelial growth factor) and its receptor, VEGFR, have been shown to play major roles not only in physiological but also in most pathological angiogenesis. Angiogenesis requires initiation by proangiogenic factors, such as VEGF, and mediated the Rho GTPases Rac-1. In addition angiogenesis via VEGF involves the main mechanism of oxidative stress [42]. VEGF also plays a pivotal role in diabetes that mediates the hyperglycemia-induced pathological effect by oxidative stress [43], which activates VEGF and IGF-1 signaling pathways via protein kinase C (PKC) and interrelated each other. VEGF is secreted by human fat cells and other tissues are stimulated by hypoxia, as well as several hormones and growth factors like insulin, IGF-1, estrogen leptin, and so on [44].

Glutathione (GSH)/glutathione disulfide is the major redox couple in animal cells and effectively scavenges free radicals and other reactive oxidative species directly and indirectly through enzymatic reactions. GHS has critical role in regulating lipid, glucose, and amino acid utilization [45]. The reduction in tissue levels of glutathione, a cellular antioxidant, increased oxidative stress markers and impaired glucose homeostasis. Increased numbers of lipid peroxidation markers have been observed in the liver of animal models of diabetes and obesity, but GSH reductase activates antioxidant defense mechanism and decreases lipid peroxidation markers [46].

Rac-1, small GTP binding protein, plays many important biological functions in cells adhesion, migration, and inflammation. Rac-1 is a mediator of VEGF signaling pathway that involves permeability and cell migration. Rac-1 is associated with adiponectin and has a direct connection with hyperglycemia and ß-cell apoptosis [47]. In addition, Rac-1 and NADPH have been reported to be a key regulator of oxidative stress through its coregulatory effects on nitric oxide synthase and NADPH oxidase [48]. In addition, Rac-1/NADPH oxidase-derived oxidative stress is involved in the pathogenesis of MetS [48] and participates in the production of cardiac hypertrophy [49]. Besides, three pathways related to OBS in our study were also involved in inflammation [2, 41], supported by our result that OBS was inversely associated with CRP and WBC. OBS may interact with gene and modify the risk of MetS via several biological pathways.

Several studies using OBS mentioned their limitations for lacking of endogenous factors that modify oxidative stress [7, 9, 33, 50]. One of the limitations of our study is that we did not consider oxidative stress induced by endogenous factors such as mitochondrial function and antioxidant enzymes or insulin signaling on MetS. However, some papers found that OBS was associated with oxidative stress biomarkers (F2-isoprostane, fluorescent oxidation products, and so on) and was predictor for incident, sporadic colorectal adenoma [9, 33]. None of the adjusted associations between hypertension and oxidative stress markers (including mitochondrial DNA copy number) were statistically significant except OBS [6]. Labadie et al. reported that* SOD2*,* CAT*, and* GSTP1* did not modify OBS even though OBS was associated risk of disease [51]. Impaired insulin signaling could be central to the development of the MetS and can promote cardiovascular diseases through abnormal lipid metabolism. Even though we did not check insulin signaling with OBS for MetS, we checked HOMA level without T2D patients and we found that HOMA level was significantly decreased by OBS levels (beta = −0.87, *p* < 0.01, data not shown). We also considered the biological process with genetic factor and oxidative stress level of OBS on MetS. Another limitation of this study is that OBS components are based on self-report data to assess dietary pro- and antioxidant exposure. FFQ is the most practical and common assessment in cohort studies. Although a 24 h diet recall survey has been conducted in this study [22], recall bias and error still remained.

## 5. Conclusion

In summary, we identified that individuals with high OBS may have lower risk of MetS. In addition, we showed interactions between genetic polymorphism and OBS through several signaling pathways. Such information would provide scientific knowledge of comprehensive biological contribution by complex exposures to pro- and antioxidants. Further research is needed to understand that modification of oxidative balance could be preventive strategy for the development of MetS.

## Figures and Tables

**Table 1:** Baseline characteristics of this study by OBS quantile^a^.

Characteristics	Q1 (*n* = 2069)	Q2 (*n* = 1169)	Q3 (*n* = 2195)	Q4 (*n* = 910)	*p* trend^c^
Age (years)^a^	57.26 ± 9.00	56.62 ± 8.84	55.24 ± 8.60	54.30 ± 8.27	<0.001
Sex					<0.001
Male	1432 (69)	569 (49)	817 (37)	199 (22)	
Female	637 (31)	600 (51)	1378 (63)	711 (78)	
Region					<0.001
Rural (Ansung)	1194 (58)	618 (53)	989 (45)	419 (46)	
Urban (Ansan)	875 (42)	551 (47)	1206 (55)	491 (54)	
BMI	24.30 ± 3.12	24.58 ± 3.12	24.67 ± 2.92	24.65 ± 3.05	0.105
Education (years)					<0.001
Elementary school or less	261 (14)	300 (12)	98 (9)	88 (9)	
Middle school graduate	814 (44)	1122 (46)	475 (44)	420 (44)	
High school or higher	770 (42)	1036 (42)	503 (47)	440 (47)	
Monthly income^b^					<0.001
<1000	724 (40)	876 (36)	318 (30)	259 (27)	
1000–2000	422 (23)	550 (23)	238 (22)	200 (21)	
≥2000	682 (37)	450 (41)	516 (48)	486 (52)	
Total energy intake^d^ (kcal/day)	1621.12 ± 471.95	1678.42 ± 519.85	1831.86 ± 589.55	2019.24 ± 848.68	<0.001

^a^Equal weight method; ^b^US dollar in 2014; ^c^*p* for linear trend was determined by the general linear model for continuous variables and by *χ*^2^ test for categorical variables. ^d^Total energy intake was calculated using food frequency questionnaires.

**Table 2:** Oxidative balance score assignment scheme.

OBS components	Score assignment scheme^a^
Iron	0 = high (3rd tertile), 1 = medium (2nd tertile), and 2 = low (1st tertile)
Vitamin C	0 = low (1st tertile), 1 = medium (2nd tertile), and 2 = high (3rd tertile)
Retinol	0 = low (1st tertile), 1 = medium (2nd tertile), and 2 = high (3rd tertile)
Carotene	0 = low (1st tertile), 1 = medium (2nd tertile), and 2 = high (3rd tertile)
Physical activity (Phy-MET)	0 = low (1st tertile), 1 = medium (2nd tertile), and 2 = high (3rd tertile)
Smoking	0 = current smoker, 1 = former smoker, and 2 = never smoker
Alcohol	0 = heavy drinker (≥50 g/day), 2 = nonheavy drinker (<50 g/day)

^a^Low, medium, and high categories corresponding to baseline tertile values among participants in the KARE cohort.

**Table 3:** Individual component level of OBS between non-MetS and MetS groups.

Characteristics	Non-MetS (*n* = 4838)	MetS (*n* = 1576)
Nutrients		
Iron, mg/day	9.74 ± 5.31	9.11 ± 4.54
Vitamin C, mg/day	103.04 ± 66.79	94.40 ± 59.82
Retinol, *μ*g/day	62.47 ± 62.16	50.98 ± 53.46
Carotene, *μ*g/day	2429.53 ± 2026.43	2412.37 ± 2030.09
Alcohol consumption, g/day	9.70 ± 22.68	9.26 ± 24.72
Nonregular drinker	4575	1470
Regular drinker^a^	248	101
Smoking status, *n* (%)		
Never	2944	1052
Former	977	238
Current	911	285
Physical activity, MET/day	82.46 ± 21.11	81.44 ± 21.36

^a^≥50 g/day of alcohol drinking; MET, metabolic equivalent.

**Table 4:** Association of the OBS with metabolic syndrome by weighing method^a^.

OBS	Number of cases (control)	OR (95% CI)	*p*	*p* trend
OBS-equal weight (AUC = 0.823)				
Quantile 1	547 (1522)	1		<0.01
Quantile 2	314 (855)	0.94 (0.78–1.15)	0.56	
Quantile 3	510 (1685)	0.81 (0.68–0.97)	0.02	
Quantile 4	186 (724)	0.65 (0.51–0.82)	<0.01	
OBS-equal weight^b^ (AUC = 0.823)				
Quantile 1	353 (1351)	1		<0.01
Quantile 2	205 (774)	0.91 (0.72–1.14)	0.40	
Quantile 3	328 (1513)	0.79 (0.64–0.97)	0.02	
Quantile 4	118 (644)	0.60 (0.45–0.81)	<0.01	
OBS-beta coefficient (AUC = 0.824)				
Quantile 1	378 (1204)	1		<0.01
Quantile 2	397 (1161)	0.65 (0.52–0.81)	<0.01	
Quantile 3	431 (1209)	0.67 (0.49–0.90)	<0.01	
Quantile 4	355 (1212)	0.56 (0.76–0.41)	<0.01	
OBS-beta coefficient^b^ (AUC = 0.829)				
Quantile 1	239 (1089)	1		<0.01
Quantile 2	272 (1047)	0.66 (0.50–0.87)	<0.01	
Quantile 3	376 (1050)	0.66 (0.47–0.91)	0.01	
Quantile 4	218 (1107)	0.56 (0.38–0.82)	<0.01	
OBS-PCA (AUC = 0.824)				
Quantile 1	375 (1203)	1 (reference)		<0.01
Quantile 2	389 (1174)	0.64 (0.51–0.79)	<0.01	
Quantile 3	419 (1189)	0.68 (0.50–0.92)	0.01	
Quantile 4	372 (1216)	0.55 (0.40–0.75)	<0.01	
OBS-PCA^b^ (AUC = 0.828)				
Quantile 1	233 (1087)	1		<0.01
Quantile 2	251 (1068)	0.71 (0.55–0.92)	<0.01	
Quantile 3	292 (1037)	0.66 (0.46–0.96)	0.03	
Quantile 4	228 (1098)	0.62 (0.43–0.91)	0.01	

^a^Adjusting for age, sex, area, and BMI. ^b^Excluded patients with type 2 diabetes; *n* = 5363.

**Table 5:** Associations of the OBS with metabolic related disorders by OBS quantile.

Characteristics	Q1 (*n* = 2069)	Q2 (*n* = 1169)	Q3 (*n* = 2195)	Q4 (*n* = 910)	*p* trend
Metabolic syndrome, *n* (%)	547 (26)	314 (27)	510 (23)	186 (20)	<0.001
Metabolic components, *n* (%)					
Abdominal obesity	686 (30)	416 (36)	753 (34)	305 (34)	0.071
Hypertriglyceridemia	748 (36)	382 (33)	631 (29)	235 (26)	<0.001
Low HDL cholesterol	1087 (53)	667 (57)	1294 (59)	539 (59)	0.005
High blood pressure	486 (24)	287 (25)	432 (20)	161 (18)	0.267
High fasting glucose	465 (23)	244 (21)	392 (18)	131 (14)	0.163
MetS score	1.68 ± 1.30	1.71 ± 1.28	1.60 ± 1.25	1.51 ± 1.25	<0.001
CRP (mg/dL)	1.66 ± 3.19	1.49 ± 2.69	1.55 ± 3.93	1.35 ± 3.18	0.013
WBC (10^3^/*µ*L)	6.70 ± 2.04	6.35 ± 1.79	6.20 ± 1.82	5.97 ± 1.71	<0.001

CRP, C-reactive protein; WBC; white blood cell count; *p* trend was assessed by *χ*^2^ test or general linear regression for linear trend.

**Table 6:** Association of the OBS with inflammatory markers^a^.

OBS	Beta coefficient	95% CI	*p*	*p* trend	Beta coefficient	95% CI	*p*	*p* trend
	CRP (mg/dL)				WBC (10^3^/*µ*L)			
Quantile 1	0	(Reference)		<0.01	0	(Reference)		<0.01
Quantile 2	−0.22	−0.30 to −0.14	<0.01		−0.77	−0.92 to −0.63	<0.01	
Quantile 3	−0.12	−0.23 to −0.01	0.04		−0.82	−1.02 to −0.61	<0.01	
Quantile 4	−0.28	−0.40 to −0.17	<0.01		−0.98	−1.19 to −0.77	<0.01	

^a^Adjusting for age, sex, geographic area, and BMI.

**Table 7:** Pathway-based analysis for interaction between OBS and genetic variation for metabolic syndrome.

Resources	Biological process	Description	*p* value	FDR	Significant genes/selected genes/all genes
KEGG Hsa04370	VEGF signaling pathway	Genes involved in VEGF signaling pathway.	<0.001	0.020	25/54/70
KEGG Hsa00480	Glutathione metabolism	Genes involved in glutathione metabolism	<0.001	0.022	12/26/39
Biocarta	Rac-1 pathway	Rac-1 is a Rho family G protein that stimulates formation of actin-dependent structures	<0.001	0.045	14/20/22
Biocarta	Rho pathway	Rac-1 is a Rho family G protein that stimulates formation of actin-dependent structures such as filopodia and lamellipodia	0.001	0.062	14/25/31
Biocarta	TNFR1 pathway	Tumor necrosis factor alpha binds to its receptor TNFR1 and induces caspase-dependent apoptosis	0.005	0.085	13/23/29
